# Identification of a disulfidptosis‐related prognostic signature for prediction of the effect of treatment in patients with endometrial carcinoma

**DOI:** 10.1002/cai2.120

**Published:** 2024-04-23

**Authors:** Lu Peng, Yuan Gao, Zifeng Cao, Yingxin Pang

**Affiliations:** ^1^ Department of Obstetrics and Gynecology Qilu Hospital of Shandong University Jinan China; ^2^ Department of Clinical Medicine Medical School of Shandong University Jinan China; ^3^ Medical Integration and Practice Center Medical School of Shandong University Jinan China

**Keywords:** disulfidptosis, endometrial carcinoma, immunotherapy, prognostic signature, tumor microenvironment

## Abstract

**Background:**

Disulfide, an essential compounds family, has diverse biological activity and can affect the dynamic balance between physiological and pathological states. A recently published study found that aberrant accumulation of disulfide had a lethal effect on cells. This mechanism of cell death, named disulfidptosis, differs from other known cell death mechanisms, including cuproptosis, apoptosis, necroptosis, and pyroptosis. The relationship between disulfidptosis and development of cancer, in particular endometrial carcinoma, remains unclear.

**Methods:**

To address this knowledge gap, we performed a preliminary analysis of samples from The Cancer Genome Atlas database. The samples were divided equally into a training group and a test group. A total of 2308 differentially expressed genes were extracted, and 11 were used to construct a prognostic model.

**Results:**

Based on the risk score calculated using the prognostic model, the samples were divided into a high‐risk group and a low‐risk group. Survival time, tumor mutation burden, and microsatellite instability scores differed significantly between the two groups. Furthermore, a between‐group difference in treatment effect was predicted. Comparison with other models in the literature indicated that this prognostic model had better predictive anility.

**Conclusion:**

The results of this study provide a general framework for understanding the relationship between disulfidptosis and endometrial cancer that could be used for clinical evaluation and selection of appropriate personalized treatment strategies.

## Graphical abstract

The role of disulfide bond in cancer has been widely studied, which is an essential regulator of extra cellular proteins. Recently published research define a new form of cell death, caused by substantial accumulation of intracellular disulfides and named as disulfidptosis. However, studies on disulfidptosis and oncogenesis, especially endometrial cancer, are lacking. Therefore, to preliminarily determine the role of disulfidptosis in endometrial cancer, we built prognostic gene signature based on public datasets and statistical algorithm. On this basis, samples were divided into groups and the immune infiltration were explored. Tumor microenvironment and tumor mutation burden were analyzed. Medicine prediction found sensitivity difference of first‐line therapy agents, which can help in making personal treatment strategies and explain the underlying reasons of sensitivity differences from patients to patients. To sum up, this is the pioneering interpretation of the relationship between disulfidptosis and endometrial cancer, providing a general framework for predicting tumor prognosis and response to chemotherapy and immunotherapy, which would be a great material for other researchers to consult.

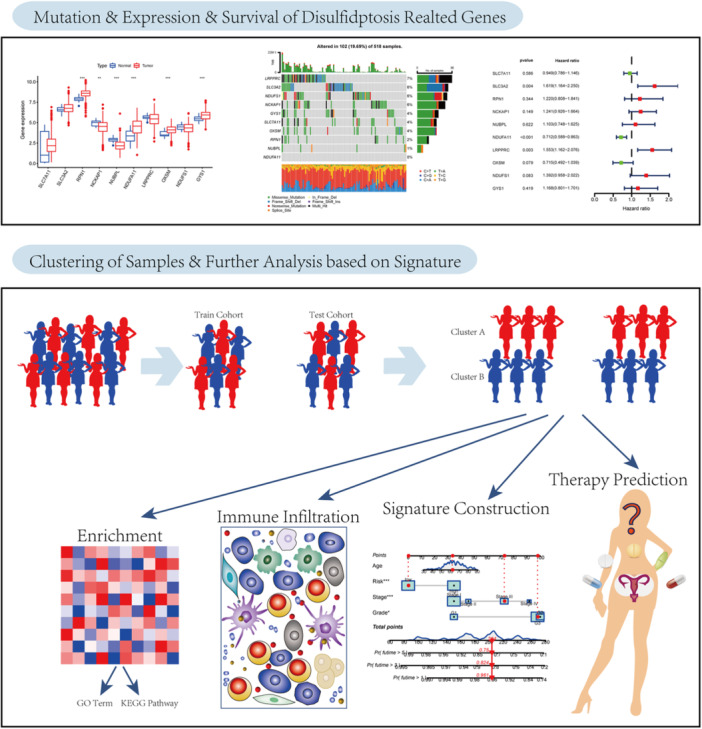

**Abbreviations**
AUC: area under the curve
CNV: copy number variation
DEGs: differentially expressed genes
dMMR: deficient mismatch repair
DRGs: disulfidptosis‐related genes
EC: endometrial carcinoma
GO: Gene Ontology
GSVA: gene set variation analysis
HR: hazard ratio
IHC: immunohistochemistry
IPS: immunophenoscore
KEGG: Kyoto Encyclopedia of Genes and Genomes
LASSO: least absolute shrinkage and selection operator
MSI: microsatellite instability
MSI‐H: microsatellite instability‐high
OS: overall survival
PCA: principal component analysis
PD1: programmed cell death 1
PDL1: programmed cell death ligand 1
PFS: progression‐free survival
ROC: receiver‐operating characteristic
SsGSEA: single‐sample gene‐set enrichment analysis
TCGA: The Cancer Genome Atlas
TMB: tumor mutation burden
TME: tumor microenvironment
Tregs: regulatory T‐cells

## 1 INTRODUCTION

Endometrial carcinoma (EC) is the most common gynecological malignancy in industrialized countries and ranks second behind cervical cancer in developing countries [1]. Despite advances in surgery and postoperative chemoradiation, there has been little improvement in survival time, and the mortality rate has continued to increase in recent years [2]. The Bokhman classification initially categorized EC into type I (estrogen‐dependent) and type II (non‐estrogen‐dependent) [3]. However, recent studies based on The Cancer Genome Atlas (TCGA) suggest that EC has four molecular subtypes with diagnostic and therapeutic implications: the POLE‐ultramutated subtype, which has the best prognosis; the microsatellite instability (MSI) subtype; the copy number variation (CNV)‐low subtype, which constitutes the majority of endometrioid cancers; and the CNV‐high subtype, which is the most aggressive type of endometrioid cancer [4]. Advances in the understanding of tumor cells and the tumor immune microenvironment have led to application of immunotherapy for various malignant tumors, including EC [5]. Immune checkpoint inhibitors (ICIs) in particular have demonstrated efficacy in patients with EC, especially for advanced, recurrent, or DNA mismatch repair deficient/MSI‐high (dMMR/MSI‐H) EC [6]. In view of the existing research, knowledge of the molecular characteristics of EC is essential for survival and prediction of the effect of treatment and patient survival.

The disulfide bond is a typical posttranslational modification that links two thiol groups in two cysteine residues [7] and is essential for the structure and function of various proteins [8]. Structural disulfides are compounds containing interdomain or intramolecular disulfide bonds, which play a key role in protecting the integrity of proteins [9]. Functional disulfides contribute to regulation of the activity, folding, and stability of proteins [10]. Abnormally marked accumulation of intracellular disulfides has an extremely toxic effect on cells [11]. Alteration of the dynamic thiol disulfide equilibrium as a result of oxidative stress is thought to be involved in the etiopathogenesis of EC [12]. A recent study found that disulfide stress is closely associated with substantial accumulation of intracellular disulfides in cancer cells accompanied by overexpression of cystine transporter solute carrier family 7 member 11 (SLC7A11), and when coupled with glucose starvation induces a new form of cell death called disulfidptosis [13]. Depletion of nicotinamide adenine dinucleotide phosphate (NADPH) is the key trigger for disulfidptosis [14]. Under normal conditions, NADPH plays an important role in counteracting disulfide stress and involves the pentose phosphate pathway. Limitation of the supply of glucose to cancer cells overexpressing SLC7A11 has been found to cause significant intracellular accumulation of cystine and collapse of the redox system, eventually leading to cell death [13]. Moreover, activation of Rac, a GTPase, and glucose transporter inhibitors can promote disulfidptosis, providing a novel target for treating cancer. *SLC7A11*, *SLC3A2*, *RPN1*, and *NCKAP1* are major suppressors and *NUBPL*, *NDUFA11*, *LRPPRC*, *OXSM*, *NDUFS1*, and *GYS1* are key promoters of disulfidptosis. However, the interaction between disulfidptosis and EC is not yet understood.

The aim of this study was to gain molecular insights into disulfidptosis and EC that have potential diagnostic and therapeutic value.

## 2 MATERIALS AND METHODS

### 2.1 Data source and selection criteria

The transcriptome profiling, single nucleotide variant, and clinical data for EC used in this analysis were downloaded from TCGA database (https://portal.gdc.cancer.gov/) and included data for 23 normal samples and 554 tumor samples. Clinical data for patients who survived less than 20 days or more than 10 years were excluded based on the assumption that survival status was related to complications or factors other than tumor itself in these patients. Another source of data, namely GSE17025 from the Gene Expression Omnibus (https://www.ncbi.nlm.nih.gov/geo/), was used to validate the results. The CNV data were downloaded from UCSC Xena (http://xena.ucsc.edu/). Key genes involved in disulfidptosis were grabbed according to the research of Liu et al. [13].

### 2.2 Clustering of EC samples

All tumor samples were clustered with the help of the K‐Means (“KM”) algorithm in the “ConsensusClusterPlus” package in R. The maximum cluster number was set as 9, and unification within the group and significant differences between groups were identified.

### 2.3 Selection of differentially expressed genes (DEGs)

With the help of the “ggpubr” and “limma” packages in R, DEGs between clusters and diseases were selected, the criterion for which was set as LogFC >1.585 with an adjusted *p*‐value of <0.05. The “VennDiagram” package in R was used to select and show the common genes.

### 2.4 Model construction

Least absolute shrinkage and selection operator (LASSO) regression based on the “glmnet” package in R was used to shrinkage genes (narrow the range of potential genes) and establish the model. The risk score was calculated for each sample using the following formula:

(1) $$\text{Risk score}=\sum_{i=1}^{n}\left({\text{Co}}_{i}\times {\text{Exp}}_{i}\right),$$

where ${\mathrm{Co}}_{i}$ is the coefficient of genes and ${\mathrm{Exp}}_{i}$ is the expression of genes.

### 2.5 Enrichment analysis

Gene set variation analysis (GSVA) was performed using the gene sets “c2.cp.kegg.v7.5.1.symbols” and “c5.go.v7.5.1.symbols,” which were downloaded from the GSEA website (http://www.gsea-msigdb.org/gsea/index.jsp). The “GSVA” and “pheatmap” packages in R were used for analysis and mapping.

### 2.6 Immune function correlation analysis

The CIBERSORT algorithm was introduced to estimate the relationship between genes and immune cells. The single‐sample gene‐set enrichment analysis (ssGSEA) algorithm was used for immune cell correlation analysis. All immunological associations were analyzed using all tumor samples. The “limma” and “ggpubr” packages in R were then used to visualize differential results. Immune function correlation analysis was performed using the same methodology. The “survival” package in R was used for analysis of survival and functions of immune‐related cells.

### 2.7 Nomogram

A nomogram that incorporated the prognostic model and basic clinical characteristics, such as age, grade, and stage, was then built to increase clinical applicability using the “regplot” and “rms” packages in R. The predictive ability of the nomogram was assessed using the area under the curve (AUC) of the receiver‐operating characteristic (ROC) curve, which is abbreviated to AUROC. The calibration curve shows the difference between the predicted value and the actual value, which is an important part of testing predictive ability, and was constructed using the calibration function within the “regplot” package in R.

### 2.8 Dimensionality reduction methods

Principal component analysis (PCA) is an algorithm that can better represent the relationship between multiple variables in large datasets, reduce data complexity, and minimize data loss [15]. The “princomp” method in the “ggplot2” package in R was used for PCA in this study.

### 2.9 Statistical analysis

Kaplan–Meier analysis and the log‐rank test were used to assess survival and to compare differences between the training and testing clusters and between risk groups. Differences were analyzed using the “limma” package in R. Survival was analyzed using the “survival” and “survminer” packages in R. All statistical analyses were performed using R software (version 4.0.3). A *p* < 0.05 was considered statistically significant.

## 3 RESULTS

### 3.1 Comprehensive landscape of disulfidptosis‐related genes (DRGs) in EC

**Figure 1:**
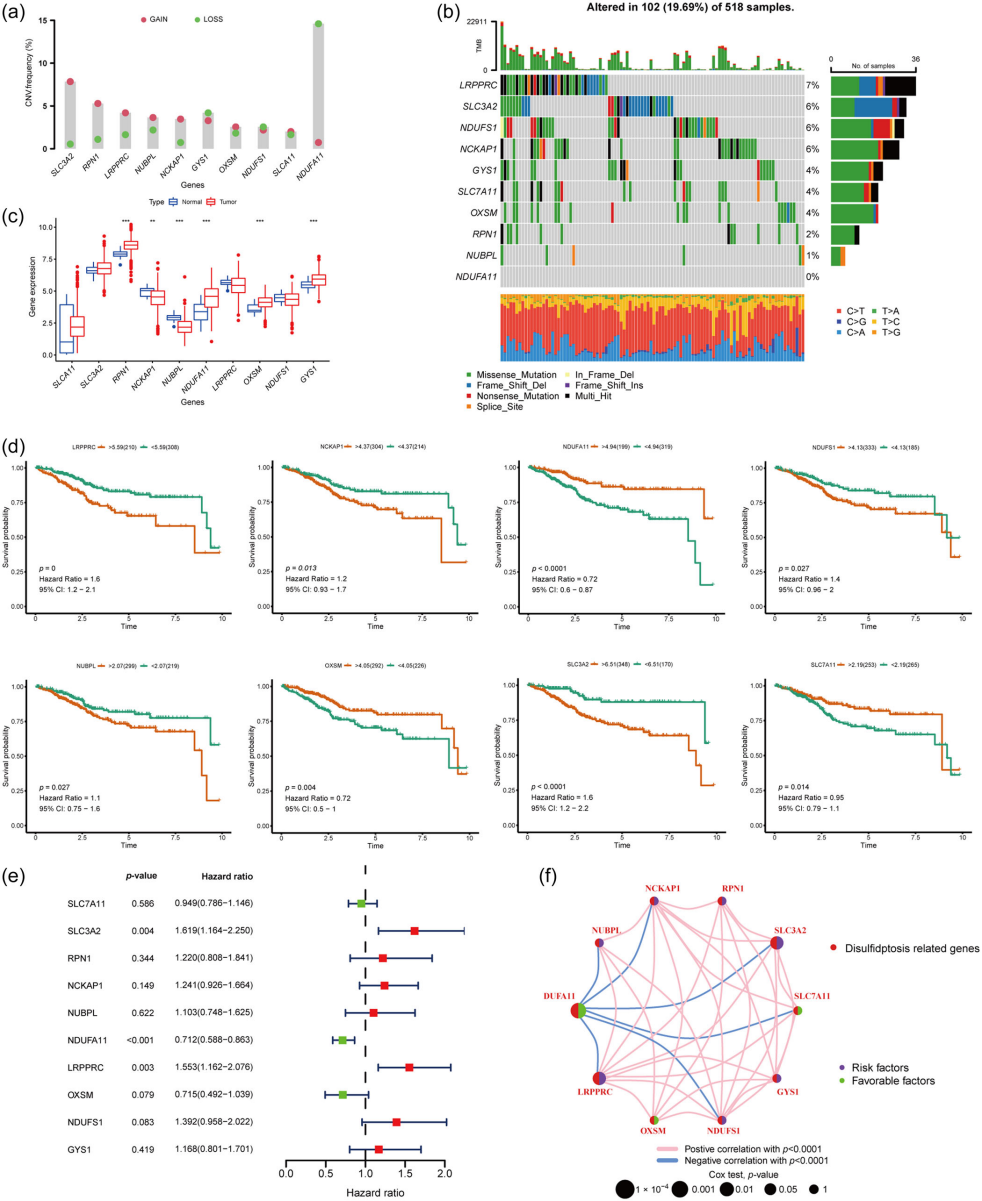
Overall landscape of DRGs in EC. (a) CNV frequency of DRGs in EC samples from TCGA datasets. The height of the bar represents the mutation frequency. Red dots means amplification of copy number while green dots is deletion of copy number. (b) Somatic mutation hot map of DRGs in each sample, containing information like the proportion of the sample with mutations, types of mutations and base changes. (c) Expression difference of DRGs between normal and tumor samples. **p < 0.01, ***p < 0.001. (d) Survival analysis of 10 DRGs in EC, 8 of which have significant difference, and are displayed. (e) Relationship evaluation of DRGs and prognosis. HR values were determined by univariate Cox analysis. HR > 1 indicates that the gene is a prognostic risk factor. (f) The interregulatory relationship between DRGs and its effect on prognosis. The color of the line represents the mode of regulation, and the size of the circle represents the effect of the gene on prognosis. CNV, copy number variation; DRG, disulfidptosis‐related gene; EC, endometrial carcinoma; HR, hazard ratio; TCGA, The Cancer Genome Atlas.

A total of 10 DRGs, namely, *SLC7A11*, *SLC3A2*, *RPN1*, *NCKAP1*, *NUBPL*, *NDUFA11*, *LRPPRC*, *OXSM*, *NDUFS1*, and *GYS1*, were analyzed. First, we plotted the CNV of the DRGs in the EC samples. *SLC3A2*, *RPN1*, *LRPPRC*, *NUBPL*, and *NCKAP1* tended to increase in copy number while *NDUFA11* tended to decrease in copy number (Figure 1a). Somatic mutations were identified in 102 (19.69%) of 518 samples; the highest mutation rate was for *LRPPRC* with no mutations found in *NDUFA11*. The most common mutation was missense mutation (Figure 1b). Expression of DRGs was also explored. Six genes exhibited significantly differential expression between normal and tumor samples; four of these genes (*RPN1*, *NDUFA11*, *OXSM*, and *GYS1*) were highly expressed in tumor tissue and the remaining two genes (*NCKAP1* and *NUBPL*) were highly expressed in normal samples (Figure 1b). The expression levels of the remaining four genes (*LRPPRC*, *SLC3A2*, *NDUFS1*, and *SLC7A11*) were validated further using the Human Protein Atlas (https://www.proteinatlas.org/), which contains immunohistochemistry data for the endometrium and EC. These online datasets showed that *LRPPRC* was more highly expressed in EC samples, while expression of *SLC3A2* and *NDUFS1* didn't express prominent difference in two specimens, and expression of *SLC7A11* was not recorded (Figure S1A). We also plotted expression of DRGs in GSE17025 (Figure S1B) and found that expression levels of *SLC7A11* and *RPN1* were higher in EC samples than in normal samples. Expression of other genes in GSE17025 showed the same trend, but none of the differences reached statistical significance. Survival analysis was performed to ascertain whether these DRGs are key to survival. Eight genes were found to be associated with a significant difference in survival status between normal samples and EC samples (Figure 1d). Higher expression of *NDUFA11*, *OXSM*, and *SLC7A11* tended to be associated with better survival, indicating a protective role in EC. Univariate Cox analysis indicated that *SLCA11* and *LRPPRC* are high‐risk prognostic genes with hazard ratios >1 while *NDUFA11* is favorable factor for survival (Figure 1e). Overall, our results indicate that these DRGs have various functions in EC. The full landscape of DRGs and their interconnections are shown in Figure 1f.

### 3.2 Cluster based on expression of DRGs

**Figure 2:**
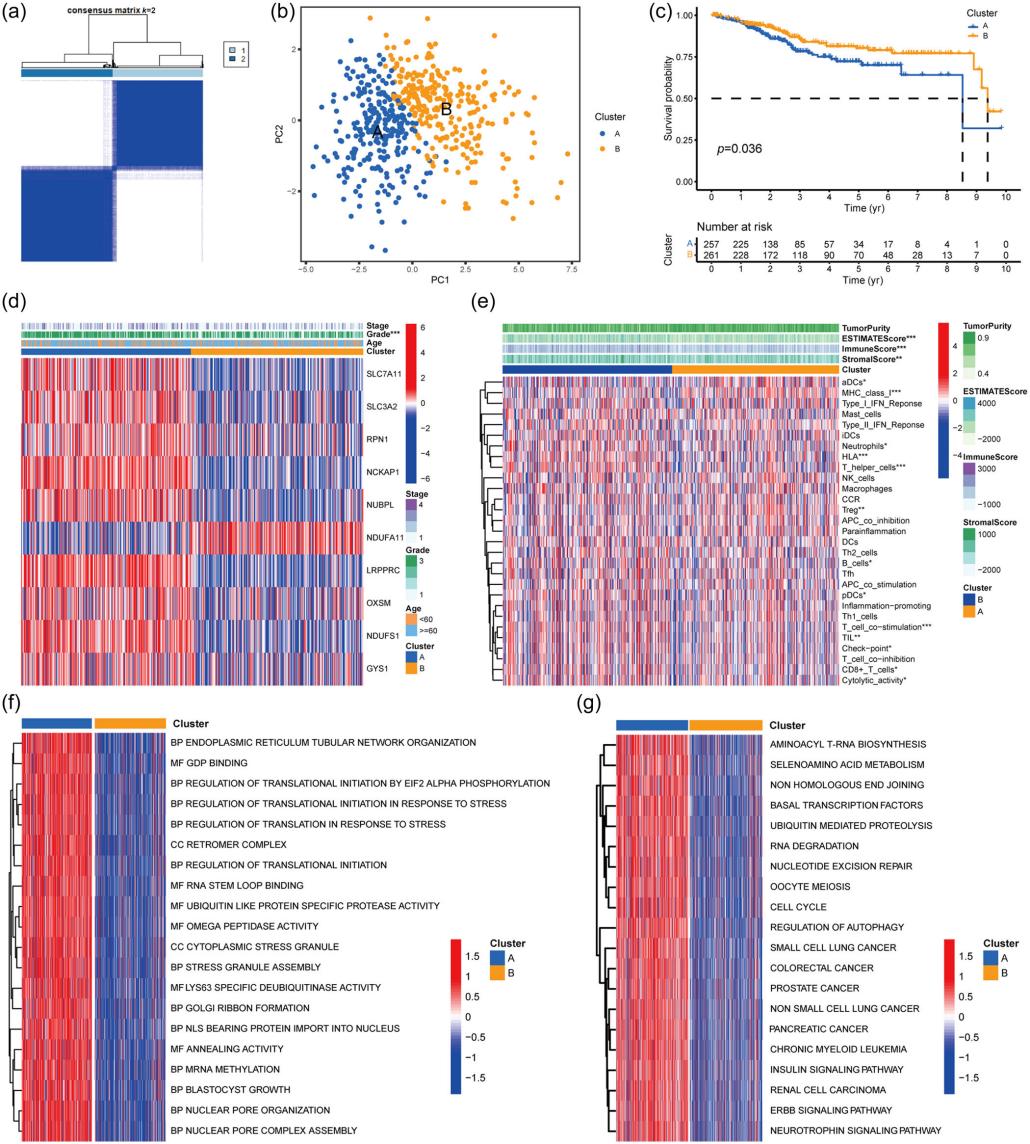
Clustering according to DRGs expression pattern. (a) With the help of “KM” algorithm, the difference was most significant when divided into two groups. (b) Separate dots cluster on PCA validate the significant difference. (c) Survival analysis of cluster A and B. (d) Hot map is used to display the clinical features and expression pattern of DRGs of every EC sample. ***p < 0.001. (e) This hot map merges the TME score and immune infiltration information of every EC sample and display the difference. *p < 0.05, **p < 0.01, ***p < 0.001. (f, g) GO and KEGG enrichment analysis conducted on cluster A and B. The top 20 terms or pathways with the most significant differences are displayed. DRG, disulfidptosis‐related gene; EC, endometrial carcinoma; GO, Gene ontology; KEGG, Kyoto Encyclopedia of Genes and Genomes; PCA, principal component analysis; TME, tumor microenvironment.

The tumor samples were divided according to the DRG expression pattern. Among the eight classification with cluster number changing from 2 to 9, samples grouped in two groups had the best effect: therefore,  the cluster number was set as 2 (Figure 2a). Separately distributed PCA dots displayed showed the prominent difference between the two clusters (Figure 2b). Survival analysis also revealed a significant difference, in that cluster B had better survival (Figure 2c), underscoring the impact of DRGs on survival. In terms of the diversity between clusters, nine of the DRGs were highly expressed in cluster A (the exception was *NDUFA11*). Patients in cluster A were more likely to have high‐grade tumors than were those in cluster B; other clinical characteristics were very similar between the groups (Figure 2d). When the tumor microenvironment (TME) score was calculated, cluster B was found to have higher stromal, immune, and ESTIMATE scores, suggesting that tumor purity was higher in cluster B. ssGSEA revealed differences in immune composition between clusters A and B. Differences in major histocompatibility complex class I, T‐helper cells, and HLA between the clusters were especially marked (Figure 2e).

Next, GSVA was performed to investigate the differences in Gene Ontology terms and Kyoto Encyclopedia of Genes and Genomes (KEGG) pathways between the two clusters. The Gene Ontology terms enriched in the low‐risk group were associated with the ubiquitin‐related molecular function and regulation process (Figure 2f). The KEGG pathways enriched in the high‐risk group are shown in Figure 2g. The RNA‐related process and ubiquitin are on the list. From the results of this analysis, it can be concluded that cluster B has a lower grade and better survival. The TME score was higher for cluster B, indicating less tumor purity. Therefore, cluster B represents the relatively benign component in EC while cluster A represents the malignant component.

### 3.3 Construction and validation of a gene signature

**Figure 3:**
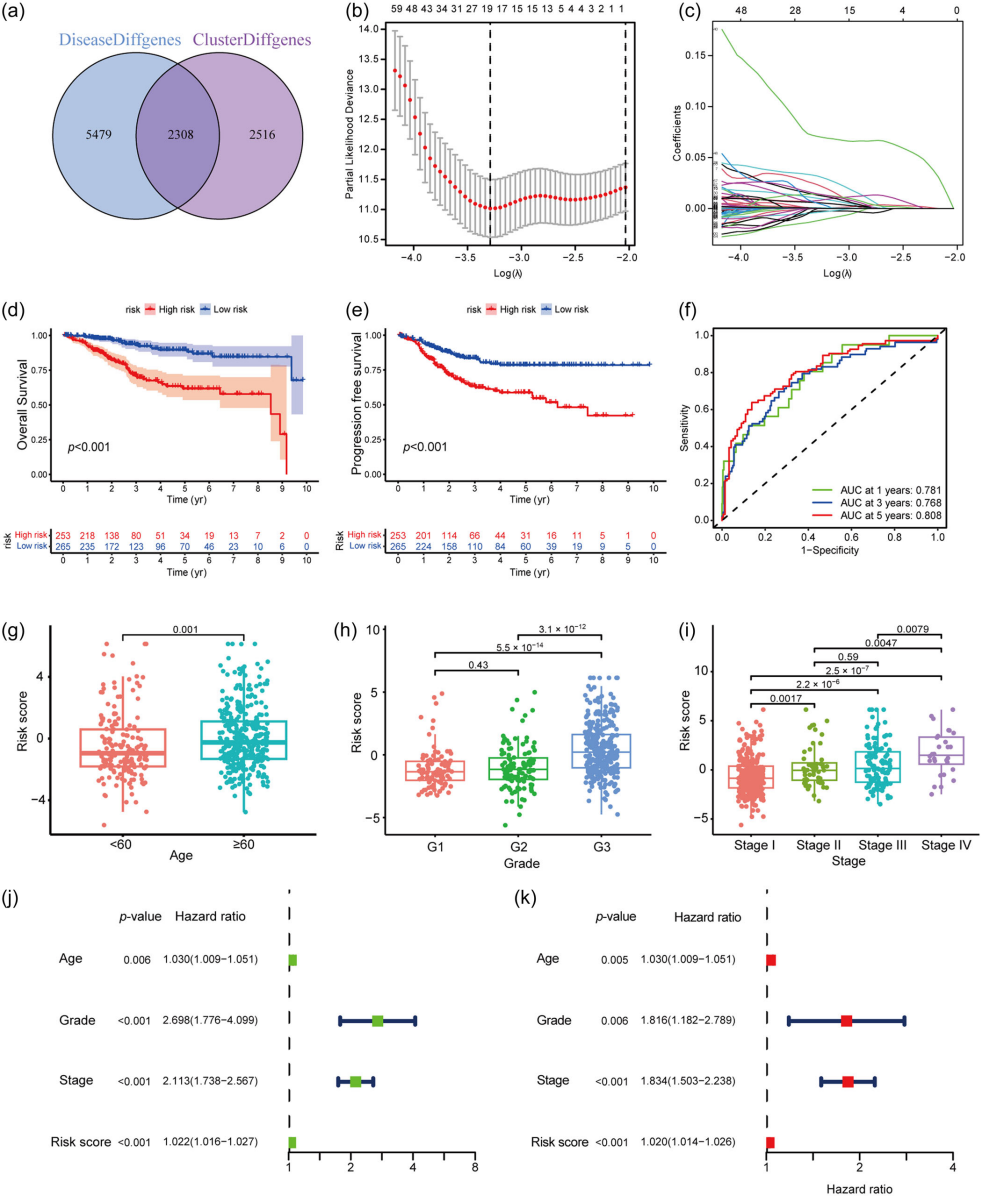
Gene signature construction and validation. (a) DiseaseDiffgenes indicates DEGs between normal and tumor samples, while ClusterDiffgenes refer to DEGs between cluster A and B. Venn diagram shows the common part of them. (b) LASSO regression analysis is used to narrow the candidate genes. (c) The correlation coefficient of each candidate genes. (d, e) OS and PFS analysis, respectively, between high‐ and low‐risk score group in all EC samples. (f) ROC curve of the signature in all EC samples. (g–i) Risk score distribute according age, grade, and stage. Age is a continuous variable and the cut‐off age is 60 as EC incidence increases after age 60. (j, k) Univariate and multivariate Cox analysis on age, grade, stage, and gene signature. DEG, differentially expressed gene; EC, endometrial carcinoma; LASSO, least absolute shrinkage and selection operator; OS, overall survival; PFS, progression‐free survival; ROC, receiver‐operating characteristic.

In total, 7787 DEGs were found between the normal sample and the tumor sample, named as DiseaseDiffgenes and 4824 DEGs between cluster A and cluster B, named as ClusterDiffgenes. The 2308 DEGs common to both samples were displayed in a Venn diagram (Figure 3a). The TCGA EC data were randomized in equal numbers to a training cohort (*n* = 258) and a testing cohort (*n* = 258). There was no significant difference in clinical features, such as age, grade, or stage, between the cohorts (Table 1). LASSO Cox regression analysis was used to identify prominent genes for construction of a gene signature in the training cohort (Figure 3b,c). Ultimately, 11 genes were selected to construct the prognostic signature. The risk score for each sample was calculated using the following formula:

(2) $$\begin{array}{l} risk\;score\\ \;=\left(\text{expression}\;\text{of}\;DIAPH2\times 0.0802815386248148\right)\\ +\;\left(\text{expression}\;\text{of}\;PRSS23\times 0.0251700746264599\right)\\ +\;\left(\text{expression}\;\text{of}\;GPAT3\times 0.0632826367474599\right)\\ +\;\left(\text{expression}\;\text{of}\;NPNT\times 0.0147101170617179\right)\\ +\;\left(\text{expression}\;\text{of}\;TMEM97\times 0.0111946963923901\right)\\ +\;\left(\text{expression}\;\text{of}\;OVOL1\times 0.0376567340000635\right)\\ +\;\left(\text{expression}\;\text{of}\;METTL21A\times 0.134746848620207\right)\\ +\;\left(\text{expression}\;\text{of}\;AC099560.2\times 0.0292382498933474\right)\\ +\;\left(\text{expression}\;\text{of}\;VTCN1\times 0.00145262623106164\right)\\ -\;\left(\text{expression}\;\text{of}\;LPCAT2\times 0.0337512404147089\right)\\ -\;\left(\text{expression}\;\text{of}\;ADH5\times 0.0170264480139817\right). \end{array}$$

**Table 1:** Differences in clinical features among train, test cohorts, and all samples.

Covariates	Type	Total, *n* (%)	Test, *n* (%)	Train, *n* (%)	*p‐*value
Age	<60	173 (33.53)	88 (34.11)	85 (32.95)	0.8521
	≥60	343 (66.47)	170 (65.89)	173 (67.05)	
Grade	Grade 1	96 (18.61)	49 (18.99)	47 (18.22)	0.973
	Grade 2	116 (22.48)	58 (22.48)	58 (22.48)	
	Grade 3	304 (58.91)	151 (58.53)	153 (59.30)	
Stage	Stage I	320 (62.02)	168 (65.12)	152 (58.91)	0.3167
	Stage II	52 (10.08)	24 (9.3)	28 (10.85)	
	Stage III	116 (22.48)	56 (21.71)	60 (23.26)	
	Stage IV	28 (5.42)	10 (3.87)	18 (6.98)	

The sample scores were classified as high‐risk or low‐risk based on the median risk score in the training group, which was 0.7102. On the basis of this cut‐off value, samples in the test cohort were classified as high‐risk or low‐risk.

There were marked differences in overall survival and progression‐free survival (PFS) between the groups in the training cohort (Figure S2A,B). Mortality increased with increasing risk score (Figure S2C), confirming a negative correlation between survival and the risk score (Figure S2D). In ROC curve analysis, the AUCs for 1, 3, and 5 years were all >0.85, indicating promising predictive ability (Figure S2E). As for validation in the test cohort, the results were consistent with those of training cohort (Figure S2F–S2J).

A marked difference in overall survival was observed when the EC samples were divided into a high‐risk group and a low‐risk group. Survival was better in the low‐risk group than in the high‐risk group (Figure 3d). PFS also demonstrated consistent conclusion (Figure 3e). The sensitivity and specificity of the signature were evaluated using ROC curves, and the AUCs for 1‐, 3‐, and 5‐year survival were 0.781, 0.768, and 0.808, respectively, indicating that the signature had reliable predictive ability (Figure 3f). The risk score was also correlated with clinical characteristics. A significant difference in risk score was observed between groups based on an age threshold of 60 years (Figure 3g). Patients aged ≥60 years tended to have higher risk scores. There was a significant difference in risk scores between grades, especially between grades 1 and 3 and between grades 2 and 3, implying a positive correlation between the risk score and a high grade (Figure 3h). The differences in distribution of risk scores between stages were also significant. It was found that the higher the risk score, the higher the stage (Figure 3i). This finding indicates a significant association between risk scores and clinical characteristics, and the positive relationship between a higher risk score and worse clinical manifestations suggests an important role of our prognostic signature in determining the severity of EC. Univariate and multivariate Cox regression analyses revealed that this signature‐based risk score is an independent prognostic factor and also a risk factor for survival, suggesting that it has potential for clinical application (Figure 3j,k).

### 3.4 Establishment of the nomogram and comparison with other models

**Figure 4:**
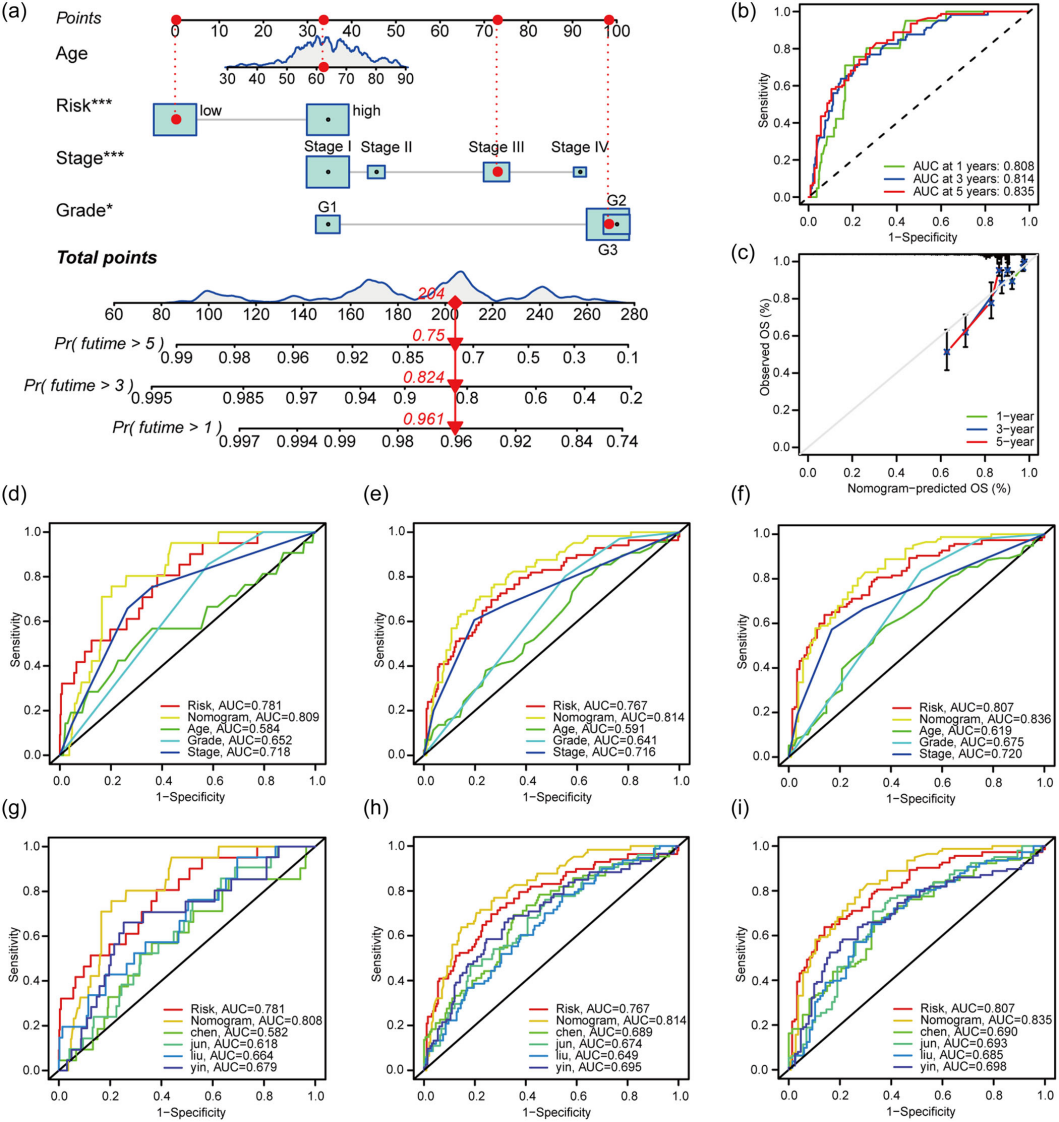
Establishment of nomogram and model comparison. (a) The weights of different indicators in the nomogram are well displayed. The red line is the score of a patient who was randomly selected as an example. *p < 0.05, ***p < 0.001. (b, c) ROC curve and calibration curve of nomogram indicates that the 1‐, 3‐, and 5‐year prediction rates are impressive and very close to the real level. (d–f) ROC curves contain nomogram, gene signature and clinical features is established to identify the prediction accuracy. (g–i) ROC curves merge different signature to compare predicting ability in 1, 3, and 5 years. ROC, receiver‐operating characteristic.

A merged scoring system was constructed to combine the gene prognostic signature with the clinical index (Figure 4a). The nomogram system assigned weights to the risk score, age, stage, and grade, and the total number of points was the sum of all scores. The total score corresponded to the predicted 1‐, 3‐, and 5‐year survival rates. ROC curves were plotted to evaluate sensitivity and specificity, and the AUCs for 1‐, 3‐, and 5‐year survival were 0.808, 0.814, and 0.835, respectively, indicating excellent predictive power (Figure 4b). The calibration curve further supported the credibility of the prediction, in that the predicted value almost coincided with the actual value (Figure 4c). Furthermore, compared with the prognostic signature and common clinical indicators, such as age, grade, and stage, the nomogram demonstrated the best predictive accuracy for 1‐, 3‐, and 5‐year survival with an AUC ≥ 0.801; the gene signature ranked second and was better than each clinical indicators (Figure 4d–f).

Many prognostic models that focus on different aspects of the development of cancer have been developed. We compared the predictive power of our prognostic model with that of existing models, including those developed by Chen et al. [16], Liu et al. [17], Weijiao et al. [18], and Zhang et al. [19]. The ROC curve indicates that the nomogram has the best predictive ability for 1‐, 3‐, and 5‐year survival, with the gene prognostic signature ranking second (Figure 4g–i). In summary, both the model based on genes and the nomogram had excellent predictive ability and potential for clinical application, suggesting an important role of disulfidptosis in EC.

### 3.5 Prediction of immunotherapy

**Figure 5:**
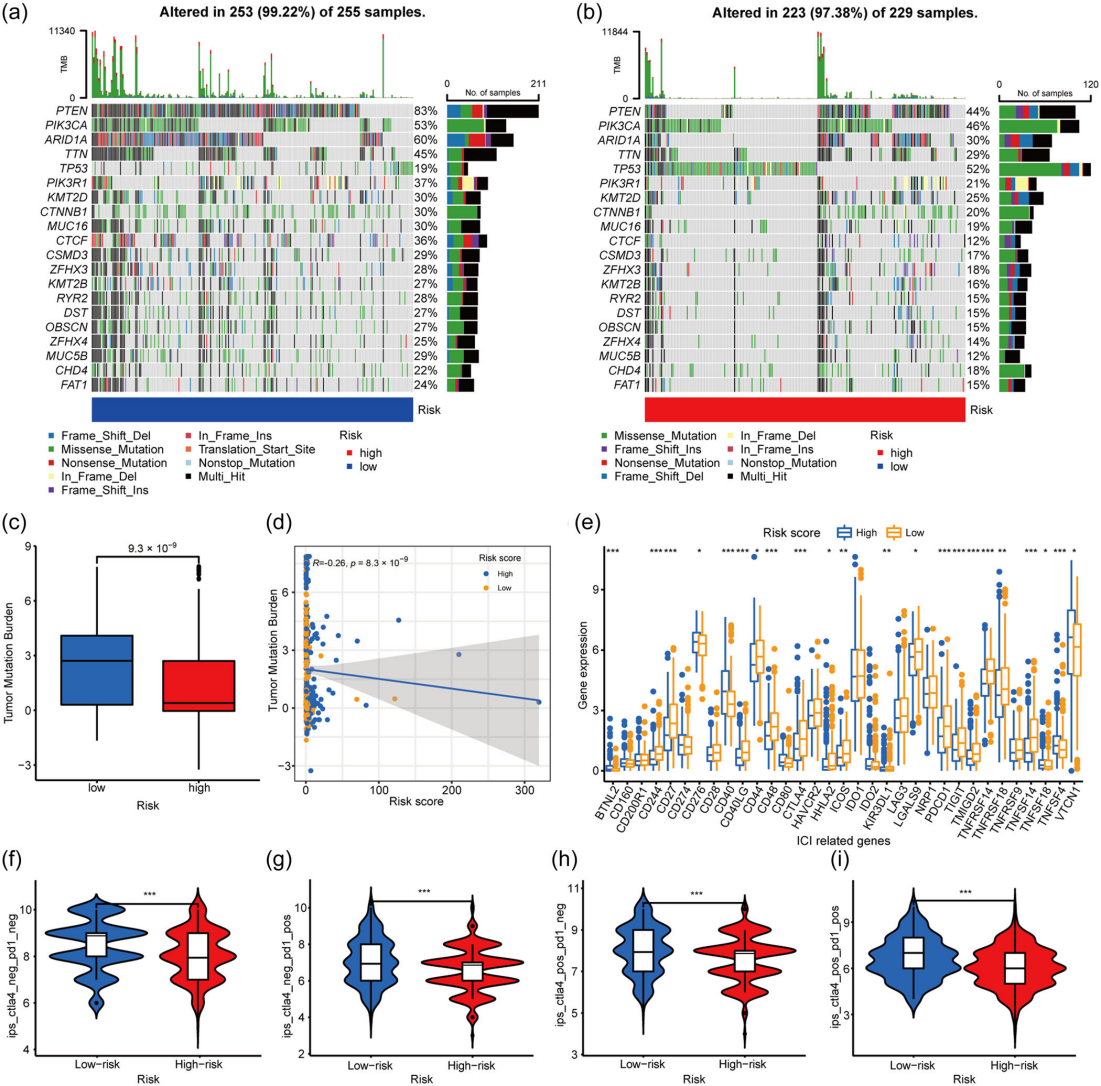
Analysis relevant to immunotherapy prediction. (a, b) Genes TMB in high‐ and low‐risk score group, respectively. The horizontal axis represents samples in each group, while the vertical axis displays the top 20 genes with the highest mutation rate. The mutation rate of each gene is marked on the right vertical axis. (c, d) Quantitative analysis of TMB and the correlation between TMB and risk score. (e) Different analysis on the expression of ICI‐related genes. (f–i) Difference analysis on IPS downloaded from TCIA. *p < 0.05, **p < 0.01, ***p < 0.001. IPS, immunophenoscore; TCIA, The Cancer Immunome Atlas; TMB, tumor mutation burden.

The somatic mutation of genes in the high‐risk and low‐risk groups was explored separately. The gene mutation percentage was much higher in the low‐risk group than in the high‐risk group; in particular, *PTEN* had 39% more mutations in the low‐risk group than in the high‐risk group. Among the 20 most highly mutated genes, only *TP53* had a higher mutation rate in the high‐risk group, with 33% more mutations than in the low‐risk group (Figure 5a,b). Quantitative analysis revealed that the tumor mutation burden (TMB) was significantly higher in the low‐risk group (Figure 5c). Cox analysis also confirmed a negative correlation of the risk score with TMB. TMB decreased as the risk score increased (Figure 5d). These findings demonstrate the relevance of risk grouping and TMB, indicating that treatment may be more effective and the survival time longer in low‐risk group after targeted ICI therapy [20].

**Figure 6:**
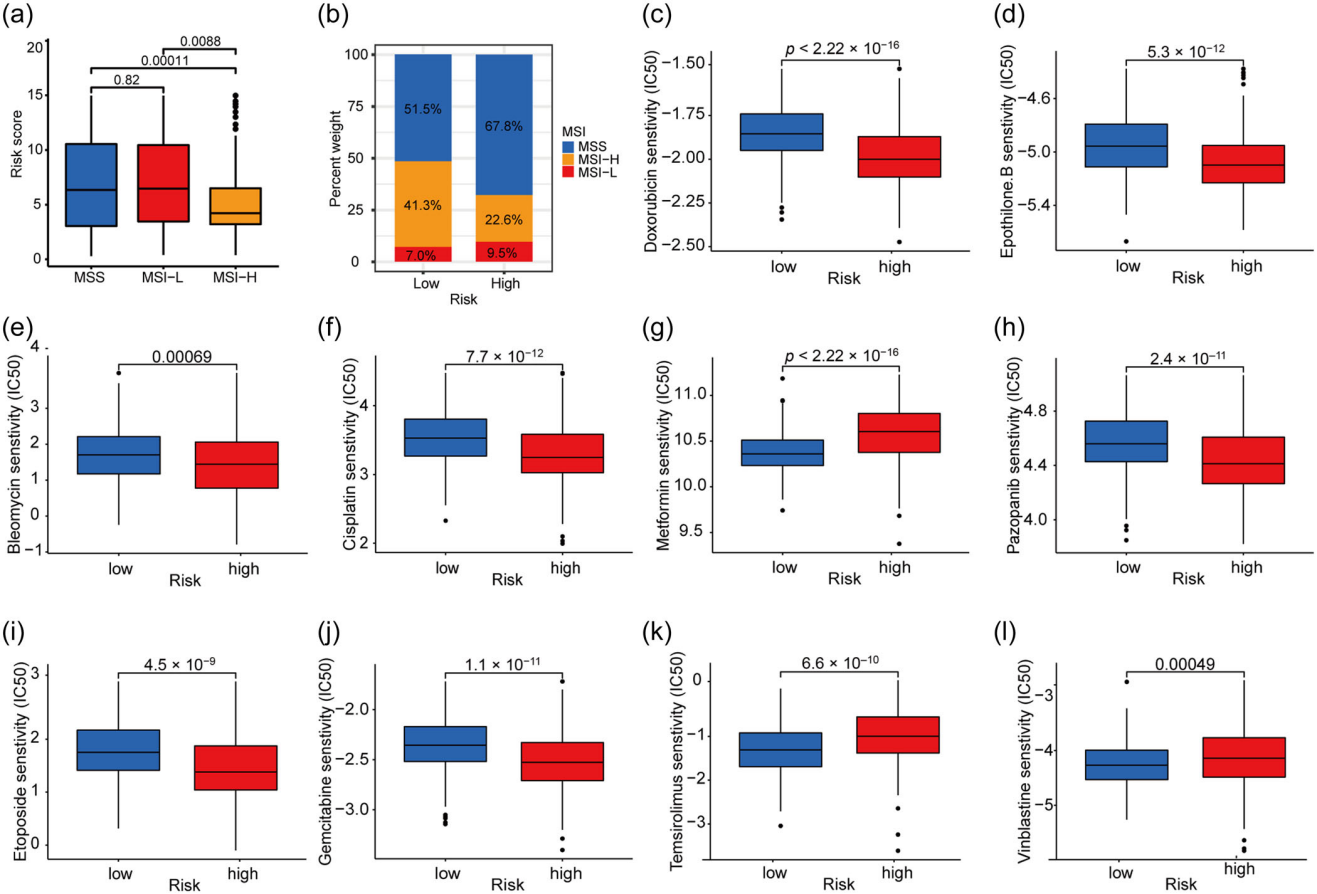
Evaluation of MSI and drug sensitivity prediction. (a) MSI score difference from TCIA. (b) Composition of MSI in high‐ and low‐risk groups. (c–l) Drug sensitivity analysis between high‐ and low‐risk score group. MSI, microsatellite instability; TCIA, The Cancer Immunome Atlas.

We then plotted and compared the expression levels of ICI‐associated genes between the gene signature groups to evaluate the relationship between the gene signature and the effect of ICI therapy. Of note is that there were significant differences in *CTLA4* and *PDCD1* expression levels between the high‐risk and low‐risk groups, with both genes being more highly expressed in the low‐risk group (Figure 5e). Next, we performed differential analysis of indicators of immunotherapy downloaded from The Cancer Immunome Atlas (TCIA, https://tcia.at/home), including the immunophenoscore (IPS), which is a quantitative immune epigenetic scoring system based on machine learning. The IPS, IPS‐CTLA4 blocker, IPS‐PD1/PDL1/PDL2 blocker, and IPS‐CTLA4‐ and PD1/PDL1/PDL2 blocker were all higher in the low‐risk group than in the high‐risk group (Figure 5f–i). Furthermore, the MSI scores from TCIA differed significantly between the high‐risk and low‐risk groups (Figure 6a). The MSI‐H samples were more likely to be in the low‐risk score group (Figure 6b). All these results suggest that the risk score can be used as an additional indicator to guide the clinical immunotherapy regimen, with a low‐risk score tending to predict a better effect of immunotherapy.

Drug sensitivity analysis using the pRRophetic package in R identified 64 sensitive agents, 10 of which are chemotherapy drugs currently in clinical use (Figure 6c–l). The IC_50_ values for bleomycin, cisplatin, doxorubicin, epothilone B, etoposide, gemcitabine, and pazopanib were higher in the low‐risk group, whereas those for metformin, temsirolimus, and vinblastine were lower in the high‐risk group. The results suggest possible options for subsequent treatment based on our scoring system.

### 3.6 Exploration of selected hub genes

**Figure 7:**
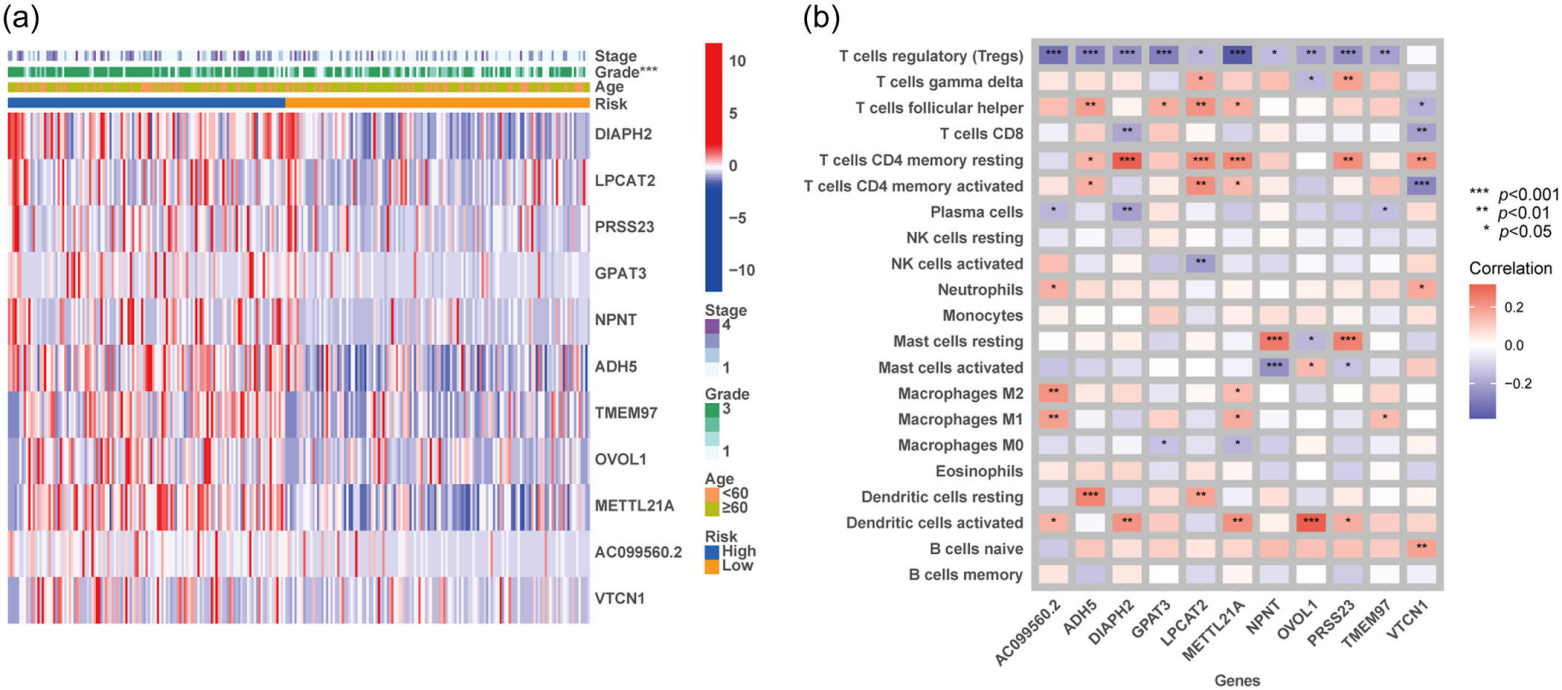
Exploration on genes that constructed the signature. (a) Intergroup expression of genes and their relationship with clinical traits. (b) Correlation of genes and immune cells according to CIBERSORT.

Screening of the hub genes that were used to construct the prognostic model from the common part of DiseaseDiffgenes and ClusterDiffgenes revealed a marked difference in expression between normal samples and tumor samples (Figure S1C). For their expressions in the GSE17025 Atlas, prominent significance had not been observed, while the different trend was consistent with that of TCGA (*p* > 0.5, Figure S1D). The heatmap showed that higher gene expression corresponded to a significantly higher disease stage and grade (*p* < 0.001) (Figure 7a). There was no obvious difference in age distribution. Furthermore, correlation of hub genes and immune cells in EC based on CIBERSORT revealed a negative relationship between regulatory T‐cells (Tregs) and these genes and a positive relationship of resting memory T‐cells and activated dendritic cells with hub genes (Figure 7b). These findings indicate that the selected hub genes play an important role in EC.

## 4 DISCUSSION

The relationship between cell death and tumorigenesis was first recognized by identification of necrosis in growing tumor cells [21]. Mutations do not cause normal cells to become tumor cells; however, cells that cannot die accumulate genetic damage that promote malignant transformation in their offspring [22, 23]. Common forms of cell death include apoptosis, ferroptosis, necroptosis, and pyroptosis [24]. Disulfidptosis is a recently recognized form of cell death, which is dependent on glucose starvation and manifests as fatal accumulation of disulfide molecules inside cells [13]. This study has shown that disulfidptosis is a potential therapeutic target in cancer including using glucose transporter inhibitors, activating Rac, and promoting overexpression of suppressors of disulfidptosis can restrain the disulfidptosis harder, which is a potential therapeutic target in cancer. However, the relationships between specific tumors and disulfidptosis remain unclear. In this study, we investigated the genes involved in disulfidptosis according to EC phenotype and immune infiltration. A gene‐related survival signature was constructed that can predict the outcome, drug application, and the immune therapeutic impact on EC.

Research on the role of genes in EC is controversial. Survival analysis of 10 DRGs gave us not only accordant directions but also some differing opinions. High expression of *LRPPRC* is associated with worse survival and poorer treatment outcomes in pancreatic cancer [25] and breast cancer [26], as is *SLC3A2* in laryngeal carcinoma [27]. The effect of genes on tumors is not consistent because of tumor heterogeneity. Unlike in previous studies of clear cell renal cell carcinoma [28] and hepatocellular carcinoma [29], we found that high *NCKAP1* expression correlated with a poor prognosis in EC. Furthermore, unlike in lung cancer [30], we identified *NDUFS1* to be a risk factor for EC. Our finding of an association between high *SLC7A11* expression and better clinical outcomes in EC was not consistent with findings in esophageal squamous cell carcinoma [31]. These inconsistent findings may reflect differences in affecting pathways and forms of induced cell death, these forms are considered to partly explain the underlying reasons for the different consequences, and more investigations in EC are required. Then, we divided our patients into two groups based on expression of 10 DRGs, divergence among which was notable. Importantly, DRGs can distinguish between relatively benign EC, indicating low‐risk score EC and better survival and malignant EC, suggesting that DRGs have a role in development and progression of this disease and could serve as prognostic indicators.

T‐cells are key players in antitumor immunity, and immunotherapy targeting T‐cells is well established. Emerging evidence suggests that infiltration of CD8^+^ T‐cells is a prognostic indicator in tumors with high TP53 mutation [32]. Other research has identified a relationship between infiltration of B‐cells and better survival in patients with high‐grade endometrioid and serous tumors [33]. We applied immune infiltration analysis based on ssGSEA and CIBERSORT to evaluate immune infiltration in EC. Our finding of more infiltration of Tregs in cluster A reflects the known ability of these cells to suppress the immune system, leading to immune escape of tumors and contributing to a poor outcome. Furthermore, we found that Tregs correlated negatively with model consisted genes, which are beneficial to survival. These findings might improve our understanding of the role of disulfidptosis in EC and its impact on prognosis and treatment.

Prediction of the prognosis and effects of treatment in patients with EC was adequate using our gene signature and nomogram but could be improved by further studies in tumor immunology and molecular biology. One study found that TP53 mutations were more common in uterine serous tumors than in endometrioid tumors [34]. The finding of a significantly higher proportion of tumors with TP53 mutations but few other gene mutations in our high‐risk group may be explained by heterogeneity in histological subtypes. Furthermore, the difference in frequency of TP53 mutation may help to guide targeted therapy. The TMB was higher in the low‐risk group than in the high‐risk group. In theory, tumor tissues with a high TMB are more easily recognized by the immune system, resulting in a good response to immunotherapy. Patients with a low‐risk score had a relatively low proportion of MSS and a high proportion of MSI‐H and showed a favorable response to PD‐1 therapy. CD276 and CD40 were highly expressed in our high‐risk group, and CD44 was overexpressed in our low‐risk group. CD276 has been found to be overexpressed on high‐grade (G3) tumors and type II carcinomas, as well as in the endothelium of the tumor‐associated vasculature [35]. The number of T lymphocytes infiltrating the tumor is also associated with expression of CD276 in cancer cells. Growth and progression of endometrial tumors may be linked to suppression of T‐cell‐mediated antitumor immunity via CD276. High CD44 expression has been associated with advanced EC, poor differentiation, greater myometrial invasion, and lymphovascular invasion [36, 37]. The reason for the different results obtained for CD44 requires further investigation. PDL‐1 and CTLA4 were found to be expressed at high levels in our study. Markedly increased expression of PDL‐1 and CTLA4 has also been reported in patients with MSI‐H colon cancer [38]. However, there is little information on expression of PDL‐1 and CTLA4 in patients with EC, and further investigations are needed. Exploration of the sensitivity to chemotherapeutic agents currently in use provided us with valuable information on application of these agents in patients with EC based on their risk score. Among the agents we selected, paclitaxel, gemcitabine, doxorubicin, and cisplatin are used first‐line for recurrence of EC [39]. In a study that included 22,632 patients, carboplatin (90.3%), paclitaxel (85.8%), cisplatin (9.4%), docetaxel (9.3%), gemcitabine (3.8%), and doxorubicin (2.0%) were identified to be the agents most frequently used as adjuvant chemotherapy [40]. In that study, 788 patients (46.8%) with recurrence received combination platinum and taxane therapy. Our analysis conducted differential grouping on transcription levels in EC and screened out sensitive therapeutic drugs in each group, which may explain why some patients had a therapeutic response to a given medication while others did not. However, this finding may have significance in terms of guiding individualized treatment and improving its effectiveness.

This study had some limitations. First, the gene signature included 11 genes. Despite the reliable predictive power of the model, evaluation of gene expression using this model may be burdensome for clinicians and add to patient expenses. Furthermore, disulfidptosis is a newly recognized phenomenon for which there have been few relevant studies. Further experiments are required to understand the relationship between disulfidptosis and EC. Nevertheless, disulfidptosis may serve as a potential therapeutic target in EC.

## 5 CONCLUSION

This preliminary study is the first to explore the association between disulfidptosis and EC. We have constructed a model that can be used to investigate the role of disulfidptosis in EC, explore immune infiltration, and screen for potential therapeutic drugs, providing new strategies for treatment of EC.

## AUTHOR CONTRIBUTIONS

**Lu Peng**: Investigation (lead); methodology (equal); software (lead); writing—original draft (equal); writing—review and editing (supporting). **Yuan Gao**: Resources (lead); writing—original draft (equal). **Zifeng Cao**: Visualization (lead); writing—original draft (equal). **Yingxin Pang**: Funding acquisition (lead); methodology (equal); project administration (lead); writing—review and editing (lead).

## CONFLICT OF INTEREST STATEMENT

The authors declare no conflicts of interest.

## ETHICS STATEMENT

Not applicable.

## INFORMED CONSENT

Not applicable.

## Supporting information

Supporting information.

## Data Availability

All data used in this experiment can be downloaded from the TCGA database and the website mentioned in this article.
