# Supplementary material for: Identification of a disulfidptosis‐related prognostic signature for prediction of the effect of treatment in patients with endometrial carcinoma
Source: Cancer Innov. 2024 Apr 23;3(3):e120. doi: 10.1002/cai2.120 (PMC11212335; doi:10.1002/cai2.120)
Supplement: Supplementary file 1 — Supporting information. [file CAI2-3-e120-s001.doc]

Fig_S1


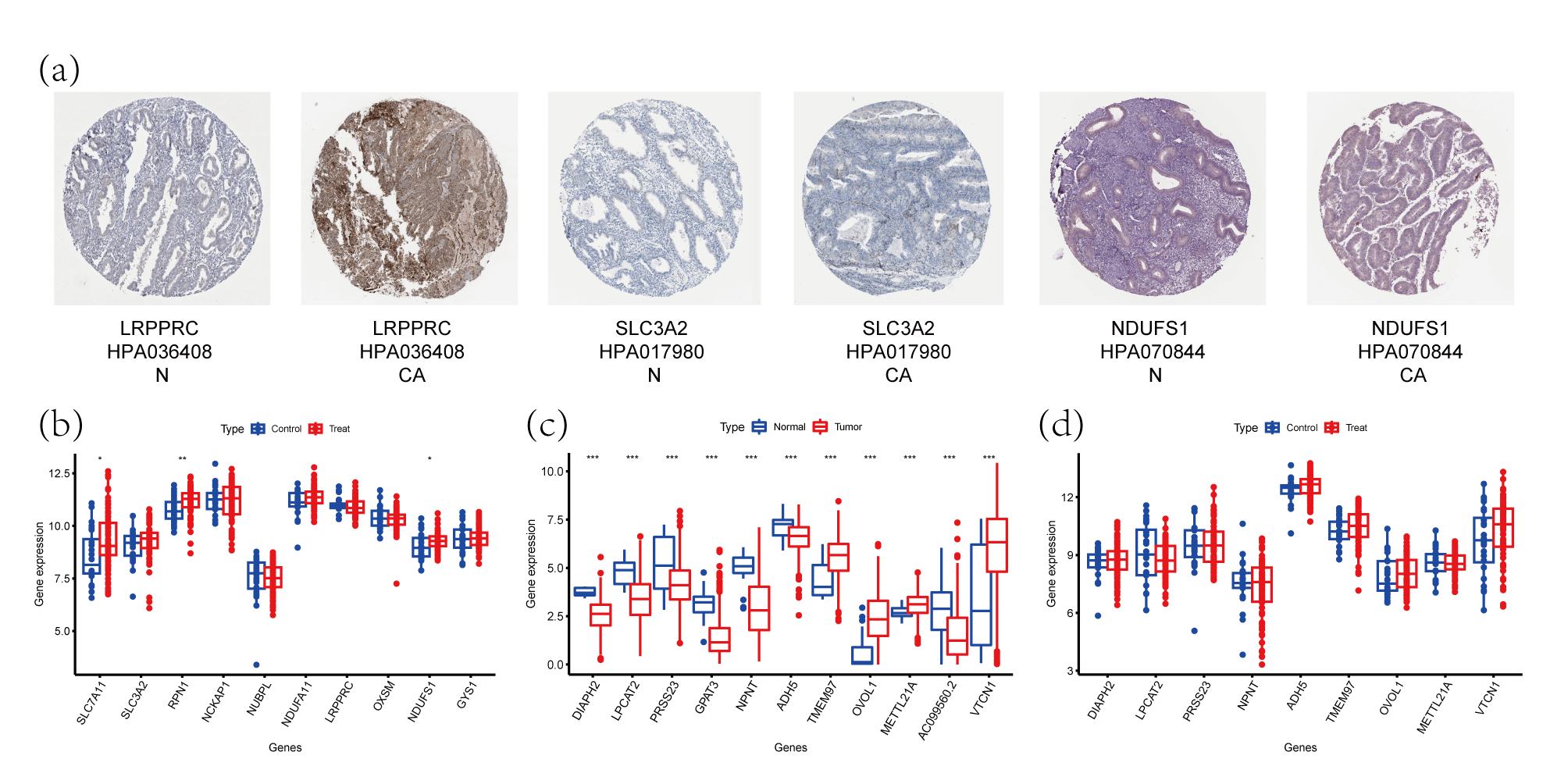


Figure S1 Expression validation of genes. (A) Gene expression in specimens. The upper lines are the name of genes. The second lines are the used antibodies of IHC. For the third lines, N indicates control groups, and CA means cancer samples. (B) Expression of DRGs in GSE17025. (C) Expression of selected hub genes in TCGA. (D) Expression validation of selected hub genes in GSE17025.

Fig_S2


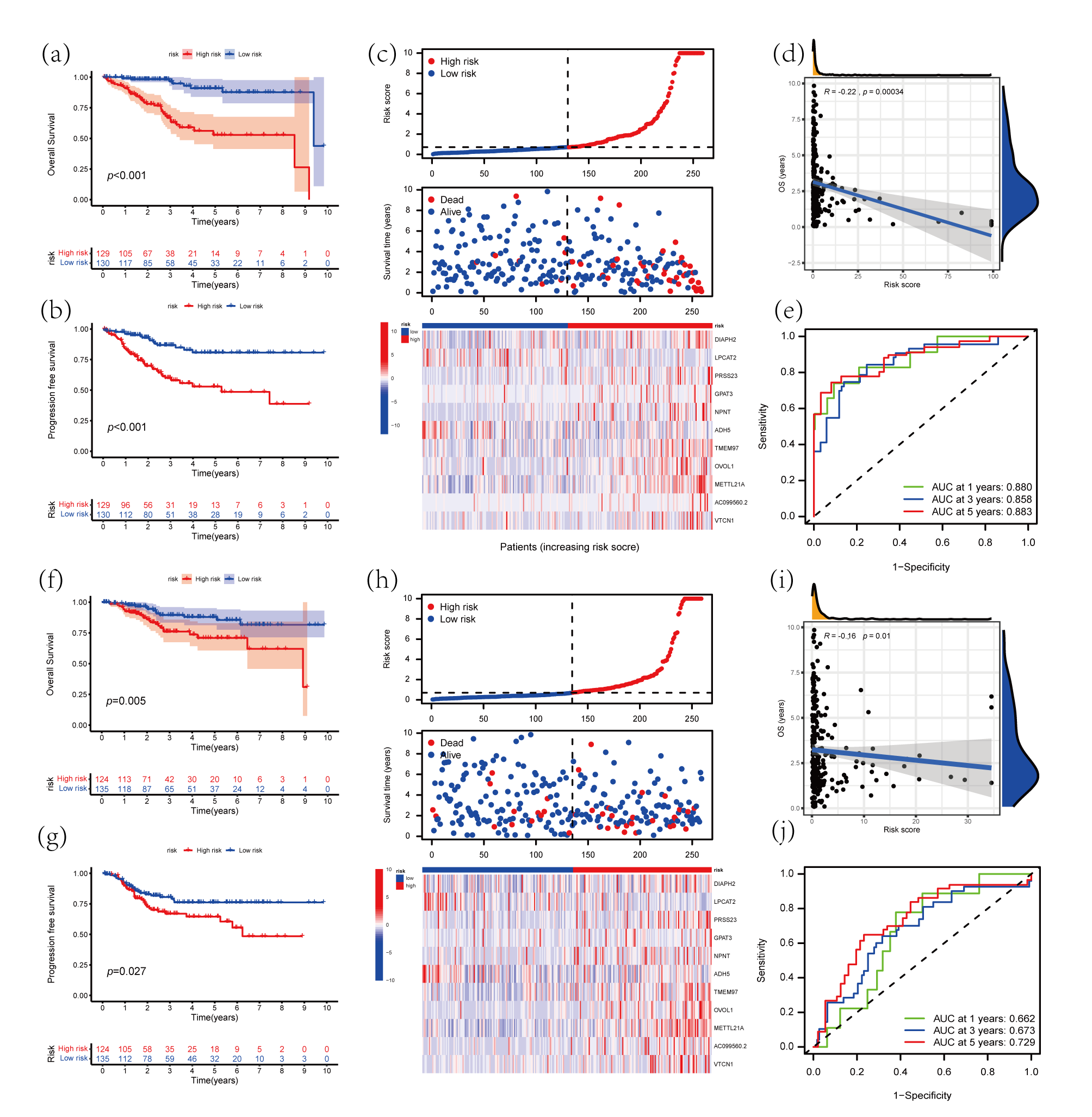


Figure S2 Survival analysis, risk curve and ROC curve in train cohort **(A-E)** and in test cohort **(F-J)**, analysis of which is the same as in all samples.
